# High sensitivity isoelectric focusing to establish a signaling biomarker for the diagnosis of human colorectal cancer

**DOI:** 10.1186/s12885-016-2725-z

**Published:** 2016-08-25

**Authors:** Narendra Padhan, Torbjörn E. M. Nordling, Magnus Sundström, Peter Åkerud, Helgi Birgisson, Peter Nygren, Sven Nelander, Lena Claesson-Welsh

**Affiliations:** 1 Department of Immunology, Genetics and Pathology, Rudbeck Laboratory, Uppsala University, Dag Hammarskjöldsv 20, Uppsala, 751 85 Sweden; 2 Stockholm Bioinformatics Centre, Science for Life Laboratory, Box 1031, 171 21 Solna, Sweden; 3 Department Surgical Sciences, Uppsala University, 751 85 Uppsala, Sweden; 4 Current address: Department of Mechanical Engineering, National Cheng Kung University, No. 1 University Road, Tainan, 70101 Taiwan

**Keywords:** Colorectal cancer, Isoelectric focusing, Signal transduction, Proliferation, ERK, c-SRC

## Abstract

**Background:**

The progression of colorectal cancer (CRC) involves recurrent amplifications/mutations in the epidermal growth factor receptor (EGFR) and downstream signal transducers of the Ras pathway, KRAS and BRAF. Whether genetic events predicted to result in increased and constitutive signaling indeed lead to enhanced biological activity is often unclear and, due to technical challenges, unexplored. Here, we investigated proliferative signaling in CRC using a highly sensitive method for protein detection. The aim of the study was to determine whether multiple changes in proliferative signaling in CRC could be combined and exploited as a “complex biomarker” for diagnostic purposes.

**Methods:**

We used robotized capillary isoelectric focusing as well as conventional immunoblotting for the comprehensive analysis of epidermal growth factor receptor signaling pathways converging on extracellular regulated kinase 1/2 (ERK1/2), AKT, phospholipase Cγ1 (PLCγ1) and c-SRC in normal mucosa compared with CRC stage II and IV. Computational analyses were used to test different activity patterns for the analyzed signal transducers.

**Results:**

Signaling pathways implicated in cell proliferation were differently dysregulated in CRC and, unexpectedly, several were downregulated in disease. Thus, levels of activated ERK1 (pERK1), but not pERK2, decreased in stage II and IV while total ERK1/2 expression remained unaffected. In addition, c-SRC expression was lower in CRC compared with normal tissues and phosphorylation on the activating residue Y418 was not detected. In contrast, PLCγ1 and AKT expression levels were elevated in disease. Immunoblotting of the different signal transducers, run in parallel to capillary isoelectric focusing, showed higher variability and lower sensitivity and resolution. Computational analyses showed that, while individual signaling changes lacked predictive power, using the combination of changes in three signaling components to create a “complex biomarker” allowed with very high accuracy, the correct diagnosis of tissues as either normal or cancerous.

**Conclusions:**

We present techniques that allow rapid and sensitive determination of cancer signaling that can be used to differentiate colorectal cancer from normal tissue.

**Electronic supplementary material:**

The online version of this article (doi:10.1186/s12885-016-2725-z) contains supplementary material, which is available to authorized users.

## Background

Although the prognosis of patients with colorectal cancer (CRC) is steadily improving, the disease remains the second most common cause of cancer-related deaths in Europe [1]. The treatment of CRC is dependent on the disease stage and the location of the tumor. Conventional treatment includes surgery, radiation and chemotherapy (5-fluorouracil, irinotecan and/or oxaliplatin) [2], often combined with bevacizumab (a neutralizing antibody against vascular endothelial growth factor; VEGF) or cetuximab/panitumumab (neutralizing antibodies against epidermal growth factor receptor; EGFR), depending on disease stage and patient-related factors [3]. During the course of CRC, mutations accumulate in genes controlling cell survival and proliferation.

Several of the genes afflicted in CRC belong to the RAS pathway [4]. The RAS pathway involves at least 4 key protein families (RAS, RAF, mitogen-activated protein kinase kinase (MEK) and extracellular regulated kinase (ERK)) that are activated in a consecutive manner, creating a signaling cascade that eventually results in gene regulation. Approximately 50 % of metastatic CRCs have activating mutations in the *KRAS* or *NRAS* genes [5–7]. Patients with *RAS* mutations do not respond favorably to treatment with neutralizing anti-EGFR antibodies [8]. BRAF is the best characterized of three closely related RAF proteins [9]. The *BRAF* gene harbors an activating mutation (V600E) in 5–12 % of all CRC [10]. Tumors may have mutations either in *KRAS* or *BRAF* though, as a rule, not in both [11]. Activation of certain protein kinase C (PKC) isoforms, such as PKCɛ, by phospholipase Cγ1 (PLCγ1), promotes RAF activation [12]. BRAF in turn activates the dual tyrosine and serine/threonine kinase MEK, which is mutated only very rarely in CRC [13]. The serine/threonine kinases ERK1/2, downstream of MEK, are also not mutated in CRC [13].

Cell proliferation is regulated also by the cytoplasmic tyrosine kinase c-SRC, which is activated when phosphorylated on tyrosine residue (Y) 418 in the kinase domain and which is inhibited when phosphorylated on the C-terminal Y527 [14]. c-SRC expression is reported to be 5–8 fold higher in premalignant colorectal polyps than in normal mucosa and a correlation between elevated c-SRC levels and CRC progression/metastatic potential has been suggested [15–17]. c-SRC kinase inhibitors are being developed for therapeutic purposes [18, 19]. Resistance to BRAF inhibition in melanoma can be overcome by inhibiting c-SRC activity [20], indicating a convergence of the pathways.

Cell survival is regulated by the phosphoinositide 3-kinase (PI3K)/AKT pathway which, via mammalian target of rapamycin complex 1 (mTORC1), eventually results in activation of p70S6 kinase and gene induction [21]. The serine/threonine kinase AKT is activated by phosphorylation of threonine (T) 308 located in the kinase domain and serine (S) 473 in the C-terminal end, by phosphatidylinositol-dependent kinase 1 (PDK1) and mTORC2, respectively. The PI3K/AKT pathway is negatively regulated by the lipid phosphatase, phosphatase and tensin homolog (PTEN) [22], which has been identified as a tumor suppressor [23]. About 15 % of all CRCs have activating or suppressing mutations in the *PI3KCA* gene, encoding the p110α catalytic subunit of PI3K, as well as the *PTEN* gene [24]. Moreover, in wild type (non-mutated) *KRAS* gene tumors, the presence of PI3K and PTEN mutations indicates a poor prognosis [25].

To identify mutations in cancer is part of an effort to individualize each patient’s treatment. However, mutations may not result in changes in protein expression levels and/or activity, and the mutation status of a particular cancer may fail to convey information about additional events occurring during progression of the disease, which may override a particular mutation, e.g. compensatory upregulation of other proteins and pathways [26]. There is no doubt that the EGFR/RAS pathway and downstream ERK1/ERK2 activities are essential in CRC etiology and disease progression [27]. However, predicting RAS pathway activity is particularly complex as there are several different upstream and parallel activators on different levels and many alternative feedback loops [26]. Apart from the regulation of RAS activity through GTPase regulatory proteins (GAPs and GEFs), downstream signaling in the RAS pathway can be induced or modulated through activities in several other pathways, including the PLCγ/PKC, PI3K/AKT and c-SRC pathways. Another complicating aspect of RAS signaling in CRC is chromosomal fragility. 85 % of sporadic CRC cases display chromosomal instability, chromosome amplification and translocation leading to aneuploidy (see [28] and refs therein), whereas the remaining 15 % of patients have high-frequency microsatellite instability phenotypes i.e. frameshift mutations and base pair substitutions [29]. The chromosomal instability of CRC clearly influences the biological consequence of the mutations. Thus, taken together, the presence of a mutation in a signaling protein does not necessarily predict activity in the corresponding signaling pathway.

Due to the existing challenges in CRC therapy, the development of rapid and sensitive screens to measure the biological activity of key signal transducers, which could serve as drug targets or as predictive or prognostic biomarkers, is warranted. Previously, the CRC proteome has been investigated using mass spectrometry to identify up- and downregulation of proteins, using mostly cell lines but also, to some extent, patient samples [30]. However, this is the first study to comprehensively address the proliferative signaling proteome in CRC tissues. For this purpose, we have developed protocols for highly sensitive, robotized isoelectric focusing, to show that signaling in the RAS pathway is dysregulated in human CRC primary tumors compared with normal mucosa. Moreover, by computational and geometric assessment of the signal transduction patterns in the different tissues examined (normal, stage II and stage IV CRC), we show that combinations of patterns from several pathways could serve as biomarkers and be exploited for the classification of tissues as normal or cancerous. We suggest that further refinement of complex signatures can be exploited for prognostic purposes.

## Methods

### Tumor biopsy collection

The colorectal tumor sampling and characterization of the anonymous samples was approved by the Uppsala Regional Ethical Review Board (no 2007/005 and 2000/001). Prior to the operation the patient was asked by the responsible surgeon to donate tumor tissue and blood samples for future molecular studies. Patients agreeing to participate were given written study information and signed an informed consent form. When the surgical specimen (colon) was removed from the patient, it was immediately transported on ice to the histopathological department and a clinical pathologist cut a 5x5x5 mm biopsy from the periphery of the primary tumor and a 10x10 mm normal mucosa more than 5 cm from the primary tumor. The biopsies were immediately placed, without addition of medium, in test tubes, which were stored at -80 °C until analyses were made. Thirty-three colon cancer samples were selected from a set of frozen tumor biopsies collected from patients operated upon for colorectal cancer at the hospitals in Karlstad or Västerås, Sweden. Seventeen of the 33 patients had stage II colon cancer and 16 had stage IV colon cancer. Samples of normal mucosa from 18 patients were available for analyses.

### Cell culture and VEGF treatment

Human umbilical vein endothelial cells (HUVECs; ATCC; Manassas, VA) were cultured on gelatin-coated 10 cm tissue culture petri dishes in endothelial cell basal medium MV2 (EBM-2, C-22221; PromoCell, Heidelberg, Germany) with supplemental pack C-39221, containing 5 % FCS, epidermal growth factor (5 ng/ml), VEGF (0.5 ng/ml), basic FGF (10 ng/ml), Insulin-like Growth Factor (Long R3 IGF, 20 ng/ml), hydrocortisone (0.2 μg/ml), and ascorbic acid (1 μg/ml). HUVECs at passages 3–6 were used. For experimental purposes, ECs were serum-starved overnight and plated in EBM-2 medium, 1 % FCS without growth factor supplement and treated with/without VEGF (50 ng/ml, Preprotech, Rocky Hill, NJ) for 7.5 min or 15 min. The cells were lysed in a commercial RIPA buffer containing protease inhibitor mix (# 040-482, ProteinSimple, Santa Clara, CA) and phosphatase inhibitors (# 040-510, ProteinSimple). The lysates were clarified by centrifugation and protein concentrations were determined by using BCA Protein Assay Kit (Pierce ThermoFisher Scientific, Rockford, IL, USA).

### Isoelectric focusing

CRC tissue samples were lysed in RIPA buffer containing phosphatase and protease inhibitors (ProteinSimple). The tissue lysates were clarified by centrifugation and protein concentration was measured by using BCA Protein Assay Kit (Pierce/ThermoFisher Scientific). Samples were run in triplicates. Lysates were mixed with ampholyte premix (# 040-972, G2 pH 5-8 or # 040-968, G2 pH 3-10) and fluorescent isoelectoric point (pI) standards (# 040-646, pI Standard Ladder 3) before being loaded into the NanoPro 1000 system (ProteinSimple) for analysis. Isoelectric focusing was performed in capillaries filled with a mixture of cell lysate (0.05–0.2 mg/ml protein), fluorescently labeled pI standards, and ampholytes. The separated proteins were cross-linked onto the capillary wall using UV light, and immobilization was followed by immunoprobing with anti-ERK1/2 (1:50, # 9102), anti-pERK1/2 (# 4377, 1:50) and anti-PLCγ1 (# 2822, 1:50) antibodies from Cell Signaling Technology (Danvers, MA); anti-AKT (# sc-8312, 1:20), p70S6 kinase (# sc-8418, 1:50), and MEK 1/2 (# sc-436, 1:50) antibodies from Santa Cruz Biotechnology Inc. (Dallas, Texas); anti c-SRC (# ab47405, 1:50) antibodies from Abcam; and anti-EGFR (# 05-484, 1:50) antibodies from Merck Millipore (Darmstadt, Germany). Analysis of HSP 70 (# NB600-571, 1:500), Novus Biologicals (Littleton, CO) was run in parallel for normalization. HRP-conjugated secondary antibodies were used, either from ProteinSimple (Goat anti rabbit-HRP IgG, # 041-081 and Goat anti mouse-HRP IgG, # 040-655 both at 1:100) or from Jackson ImmunoResearch (West Grove, PA) (Donkey anti-Rabbit IgG, # 711-035-152 and Donkey anti-Mouse-HRP IgG # 711-035-150, both at 1:300), to detect the signal. In some cases, signal amplification steps were employed by using an amplified rabbit (# 041-126, 1:100) or amplified mouse (# 041-127, 1:100) secondary antibody detection kit (ProteinSimple). The signal was visualized by enhanced chemiluminescence (ECL) and captured by a charge-coupled device (CCD) camera. The digital image was analyzed and peak area quantified with Compass software (ProteinSimple). The peak area of the protein of interest was normalized to the area of heat shock protein 70 (HSP70) in the sample, analyzed in parallel.

### Lambda phosphatase digestion

Some samples were enzymatically dephosphorylated by incubating 8–15 μg of cell lysate with 50 units of lambda phosphatase (# 14-405; Upstate Biotechnology, Charlottesville, VA), for 5-30 min at 30 °C, where incubation time was titrated independently for each signaling component. Digested samples were subjected to immunoblotting or isoelectric focusing as described above.

### Mutation analysis

*KRAS* pyro-sequencing mutational analysis was performed according to the manufacturer’s protocol for the PyroMark™ Q24 KRAS Pyro kit (QIAGEN GmbH, Hilden, Germany) and the use of PCR primers previously described for *KRAS* codon 12/13 [31], codon 61 [32], and for *BRAF* codon 600 [31]. Ten ng genomic DNA from the patients tumor tissue was used for each PCR reaction. Twenty μl PCR product was then subjected to Pyro-sequencing analysis using Streptavidin Sepharose High Performance beads (GE Healthcare, Chicago IL), PyroMark Gold Q96 reagents, PyroMark Q24 2.0.6 software, and a Q24 instrument (QIAGEN). Sequencing primer for *KRAS* codon 12/13 was 5′-AACTTGTGGTAGTTGGAGCT-3′, for codon 61 5′-TCTTGGATATTCTCGACACAGCAG-3′, and for *BRAF* codon 600 5′-TGATTTTGGTCTAGCTACA-3′. Due to sub-optimal DNA quality, two samples were not suitable for mutation analysis (denoted “unclear” in the figures).

### Immunoblotting

Ten μg of CRC tissue- or cell lysate was mixed with lithium dodecylsulfate sample buffer and Sample Reducing Agent and heated at 70 °C for 10 min. The proteins were resolved on NuPAGE Novex 4–12 % Bis-Tris SDS PAGE Gel (Life Technologies, Carldsbad, CA) and transferred onto PVDF membranes (Immobilon-P IPVH00010; Merck Millipore). The membranes were blocked by using 5 % (w/v) nonfat dry milk/BSA in TBS with 0.1 % Tween 20 for 1 h at RT, which was followed by incubation over night at 4 °C with primary antibodies pERK 1/2 (# 4377, 1:1000), ERK1/2 (# 9102, 1:1000), SRC pY416 (# 2101, 1:1000), SRC pY527 (# 2105, 1:1000), pAKT (# 4060, 1:1000), AKT (# 9272, 1:1000), PLCγ1 (# 2822, 1:1000), all from Cell Signaling Technology. SRC (# ab47405, 1:1000) and β2M (# ab75853, 1:2000) were from Abcam. EGFR (# 05-484, 1:2000) and GAPDH (# MAB374, 1:1500) from Merck Millipore, α-Tubulin (# T9026, 1:1000) from Sigma-Aldrich (Saint Louis, MI), p70S6 kinase (# sc-8418, 1:2000) from Santa Cruz Biotechnologies Inc, HSP 70 (# NB600-571, 1:1000) from Novus Biologicals. Proteins of interest were detected with HRP-conjugated donkey anti-rabbit IgG antibody (# NA934, 1: 15000) or sheep anti-mouse IgG antibody (# NA931, 1: 15000), visualized with using ECL Prime (# RPN2232) and exposed to either the Hyperfilm ECL (# 28906837) all from GE Healthcare. Signals were visualized using the ChemiDoc™ MP Imaging System (Bio-Rad Laboratories, Herkules, CA) according to the provided protocol.

All antibodies used for the isoelectric focusing were tested for specificity by immunoblotting of HUVEC lysates (for AKT, p70S6 kinase, PLCγ1, c-SRC, SRC pY527, ERK1/2, HSP 70 and MEK 1/2) and lysates from A431 cells (#12-302, Merck Millipore) for EGFR (see Additional file 1: Figure S1). Certain antibodies, such as the anti-c-Src antibodies were also validated elsewhere for example at the MD Anderson Functional Proteomics resource (RPPA core facility, see https://www.mdanderson.org/research/research-resources/core-facilities/functional-proteomics-rppacore/antibody-information-and-protocols.html.

### Statistical analysis

The Mann-Whitney *U* test was used to calculate two-tailed *p*-values of the null hypothesis that the populations of the two compared features (proteins) are the same. *p* < 0.05 was considered statistically significant. *, *p* < 0.05; **, *p* < 0.01; ***, *p* < 0.001 and ****, *p* < 0.0001. The Mann-Whitney test is a conservative, non-parametric test that was chosen to preclude false detections arising from assumptions of data distribution.

### Identification of tissue signatures

For assessment of data sets and the creation and evaluation of convex hulls for classification of the tissue samples based on signatures, see Additional file 1: Figure S3, Characteristics of the data set and errors.

## Results

### Regulation of EGFR expression and activity in CRC

**Fig. 1:**
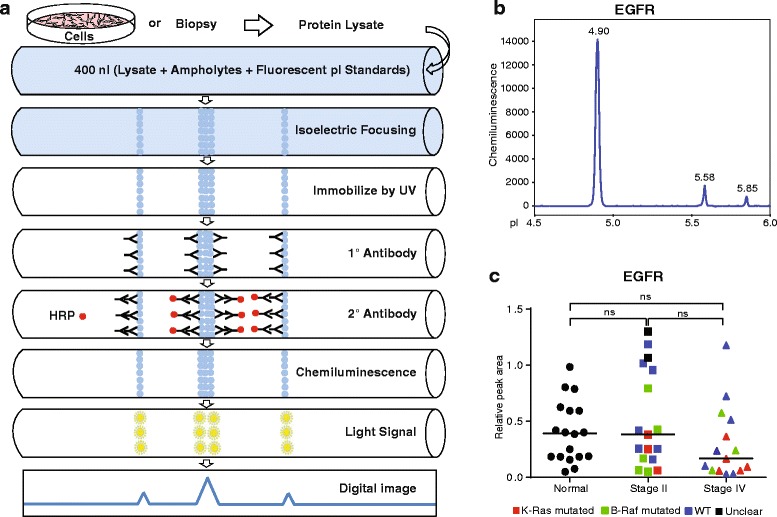
Sensitive isoelectric focusing of EGFR in normal mucosa and CRC. **a.** Schematic outline of the isoelectric focusing procedure. 400 nl of protein lysates from cultured cells or tissues are passed through the capillaries, followed by probing with antibodies and detection using ECL, resulting in an electropherogram. **b.** Representative electropherogram showing EGFR protein peaks. **c.** Plot of individual HSP70-normalized peak areas from EGFR electropherograms on normal mucosa or CRC samples. Symbols in plots indicate the mutation status of CRC biopsies: Red; *KRAS* mutated, green; *BRAF* mutated, blue; wild type (WT) with regard to *KRAS* and *BRAF*, black; unidentifiable (unclear) for *KRAS* and *BRAF*

Whereas activating mutations in the EGFR gene are rare in CRC, protein levels may be increased as a result of gene amplification or through other mechanisms e.g. involving increased translation or decreased internalization and degradation. We used isoelectric focusing for sensitive and high-resolution detection of EGFR expression in tissues, comparing normal mucosa (18 samples) with CRC samples (17 samples from stage II and 16 samples from stage IV). The mutation status of the CRC samples was determined for *KRAS* and *BRAF.* Tissues were lysed and, in a robotized procedure, proteins were immobilized to the wall of thin capillaries using UV exposure, followed by incubation with primary and secondary antibodies and ECL-detection, as outlined schematically in Fig. 1a. Tissue lysates and antibodies were loaded at desired concentrations in 384-well plates placed under the capillary holder in the instrument. As shown in Fig. 1b and c, there was no significant difference in the expression levels of EGFR when comparing normal tissue with stage II and IV CRC using isoelectric focusing, although the median was numerically lower in stage IV samples. The peaks corresponding to antibody detection of EGFR were normalized to those of HSP70 run in parallel. There was no correlation between EGFR levels and the *KRAS* or *BRAF* mutation status, in this analysis.

### Regulation of AKT and p70S6K pathways in CRC

**Fig. 2:**
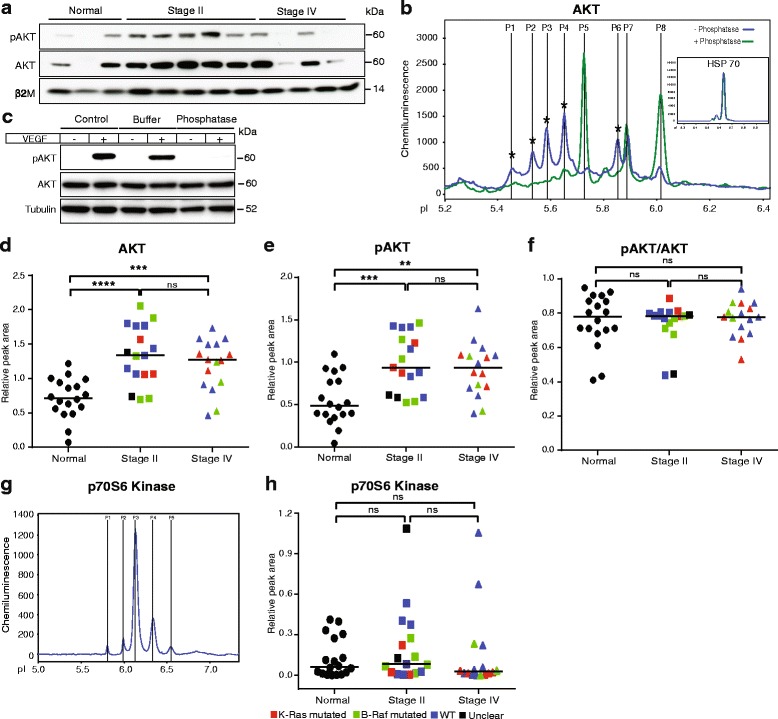
Detection of total AKT protein and phospho-protein by isoelectric focusing. Plots (**d-f, h**) show values after normalization to HSP70 levels analyzed in parallel in each sample. Symbols in plots: Red; *KRAS* mutated, green; *BRAF* mutated, blue; wild type (WT) with regard to *KRAS* and *BRAF*, black; unclear for *KRAS* and *BRAF*. **a.** Immunoblotting of selected tissue samples with antibodies against pAKT (AKT pS473) and total AKT protein. Blotting for β2 microglobulin (β2M) was used as a loading control. **b.** Representative electropherogram showing phosphorylated and non-phosphorylated AKT peaks. Blue and green lines indicate electropherograms of samples digested (green) or not (blue) with lambda phosphatase. Inset; electropherogram showing HSP70 run in parallel. **c.** Immunoblotting of HUVEC (±VEGF for 15 min) cell lysate with antibodies against pAKT and total AKT protein. Blotting for tubulin was used as to monitor equal loading. Control; without any incubation; Buffer; control lysate incubated with buffer, Phosphatase; lysate incubated with lambda phosphatase enzyme. **d.** Plot of total AKT peak areas (P1–P8) in the different samples. **e.** Plot of pAKT peak areas (present before but not after lambda phosphatase treatment; P1–P4 and P6). **f.** Plot of the ratio of pAKT/AKT peak areas. **g.** Representative electropherogram of p70S6 kinase expression. **h.** Plot of p70S6 kinase expression normalized to HSP70

Signaling in the PI3K/AKT pathway results in downstream activation of mTOR and p70S6 kinase and ultimately, cell survival and proliferation [33]. The level of AKT expression and activity was first analyzed by immunoblotting on normal mucosa and CRC samples (Fig. 2a). The level of AKT pS473 was elevated in stage II CRC, but the variability was considerable in this conventional analysis. Isoelectric focusing followed by detection of AKT resulted in a reproducible pattern with several peaks, when probed with an antibody against total AKT proteins, AKT1, AKT2 and AKT3 (Fig. 2b). The pattern of AKT-peaks was reminiscent but not identical to that described in previous reports where isoelectric focusing was used to investigate the in vitro regulation of the AKT pathway in cell lines from breast cancer and acute myeloid leukemia [34, 35].

Phospho-specific AKT antibodies did not permit specific detection of protein species in the isoelectric focusing (data not shown). Through lambda phosphatase digestion, however, several pAKT isoforms were identified (Fig. 2b; P1-4 and P6), possibly representing distinct AKT family members phosphorylated on different residues. The optimal conditions for lambda phosphatase digestion were determined by immunoblotting of phosphatase-treated control (HUVEC) cell lysates, which showed that the phosphatase treatment resulted in phosphate stripping without digestion of protein (Fig. 2c). Of note, the levels of pAKT and total AKT as detected in the capillary isoelectric focusing were significantly higher in the CRC tissues compared with normal mucosa (Fig. 2d and e). The ratio of pAKT/AKT did not change, however, indicating that the relative AKT phosphorylation level was not affected by the disease (Fig. 2f). The protein level of p70S6 kinase, a serine/threonine kinase activated downstream of PI3K/AKT, was similar in the cancer samples as compared to normal mucosa (Fig. 2g-h).

### Upregulation of PLCγ1 protein in CRC stage II and stage IV

**Fig. 3:**
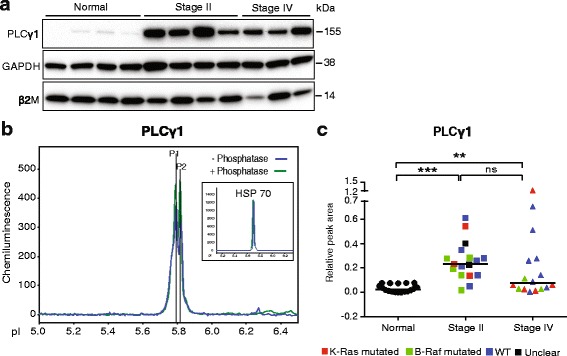
Detection of PLCγ1 total protein by isoelectric focusing. **a.** Immunoblotting of selected tissue samples with antibodies against PLCγ1. Blotting for GAPDH and β2 microglobulin (β2M) were used as loading control. **b.** Representative electropherogram showing PLCγ1 total protein peaks. Inset; electropherogram showing HSP70 run in parallel. **c.** Plot of PLCγ1 peak areas in samples from normal tissue, CRC stage II and IV biopsies. Values were normalized to HSP70 levels. Symbols in plots: Red; *KRAS* mutated, green; *BRAF* mutated, blue; wild type (WT) with regard to *KRAS* and *BRAF*, black; unclear for *KRAS* and *BRAF*

PLCγ1 is known to activate the RAS pathway to promote cell proliferation via PKC. Conventional immunoblotting for PLCγ1 allowed detection of a very faint band in the tissue lysates of normal samples while CRC stage II showed a prominent upregulation of PLCγ1 protein. In CRC stage IV samples, the signal was slightly lower (Fig. 3a). Capillary isoelectric focusing resulted in two very closely migrating peaks (Fig. 3b) which were both resistant to lambda phosphatase treatment. Antibodies against phosphorylated PLCγ1 failed to yield a signal in the isoelectric focusing (data not shown). Quantification of the combined areas of the two peaks showed a significant increase in PLCγ1 expression in stage II and IV samples (Fig. 3c), in agreement with the immunoblotting data. Moreover, the variability in expression level was higher in the cancer samples than in the normal tissue biopsies. Combined, these data indicate that while total PLCγ1 was upregulated in CRC, there was low or no accumulation of phosphorylated PLCγ1.

### Decreased c-SRC phosphorylation in CRC

**Fig. 4:**
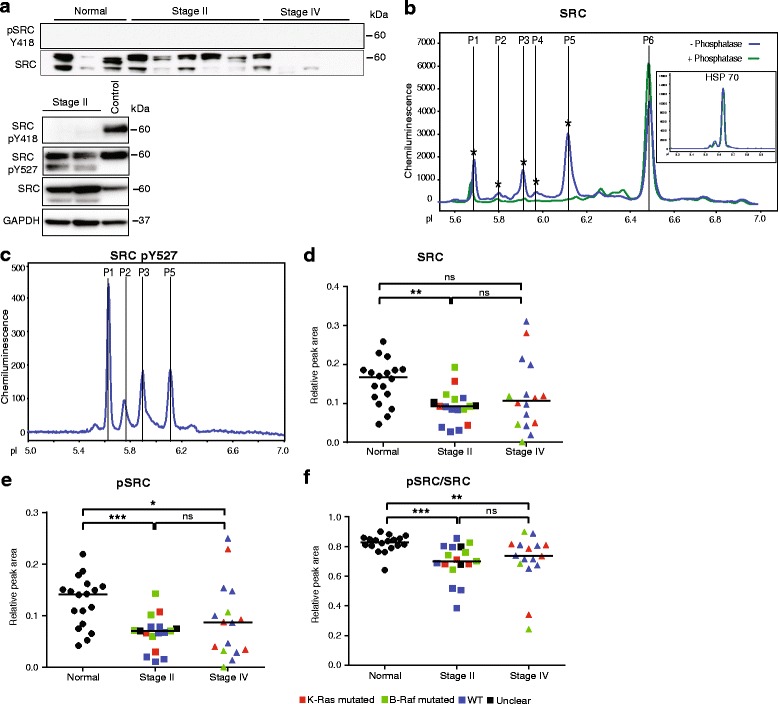
Detection of c-SRC total protein and phosphorylated forms by isoelectric focusing. Plots (**d**–**f**) show values after normalization to HSP70 levels. Symbols in plots: Red; *KRAS* mutated, green; *BRAF* mutated, blue; wild type (WT) with regard to *KRAS* and *BRAF*, black; unclear for *KRAS* and *BRAF*. **a.** Immunoblotting of selected tissue samples with antibodies against c-SRC pY418, c-SRC pY527 and total SRC protein. For upper panel, loading control β2M was same as Fig. 2a. GAPDH was used as a loading control for lower panel. Control; HUVEC cell lysate was used as a positive control. **b.** Representative electropherogram showing c-SRC total protein and phosphoprotein peaks. Phosphorylated peaks (blue line) were identified by virtue of their sensitivity to lambda phosphatase digestion (green line). Inset; electropherogram showing HSP70 run in parallel. **c.** Representative electropherogram showing c-SRC pY527 peaks. **d.** Plot of combined c-SRC peak (P1–P6) areas in normal mucosa, stage II and stage IV CRC. **e.** Plot of phosphorylated c-SRC peak (P1–P5) areas. **f.** Plot of the ratio pSRC/SRC

We also investigated the expression and activity of c-SRC, as its activity results in the downstream induction of several signaling pathways regulating cell proliferation. An antibody against total c-SRC detected several species upon immunoblotting of normal and stage II samples. In contrast, stage IV samples showed very faint or no expression of c-SRC (Fig. 4a). Moreover, all samples lacked reactivity with antibodies against c-SRC pY418, indicating low or no c-SRC activity in the colon (Fig. 4a, upper panel). Control immunoblotting of lysates from growth factor stimulated cells verified that the anti c-SRC pY418 antibodies recognized the expected 60 kDa species (Fig. 4a, lower panel). Moreover, immunoblotting with antibodies against the inactivating c-SRC pY527 residue revealed prominent bands in both the control cell lysate and in selected CRC samples (Fig. 4a, lower panel). Thus, conventional immunoblotting for total c-SRC and the phosphorylated variants showed a complex and variable pattern.

Isoelectric focusing detected six major c-SRC species (Fig. 4b); five peaks with a more acidic isoelectric point disappeared with lambda phosphatase digestion and were collected in one peak with a more basic pI of 6.5 (Fig. 4b). Probing with the c-SRC pY527 antibodies showed that the majority of the pSRC species in peaks (P)1-3,5 contained phosphorylation at the inactivating Y527 (Fig. 4c). The various pY527 antibody-reactive phosphospecies focusing at different pI may correspond to c-SRC variants with different posttranslational modifications such as serine/threonine phosphorylation [36]. We can not exclude that certain molecular species may correspond to c-SRC related proteins, containing highly similar epitopes. However, the normalized peak areas for all peaks (Fig. 4d, denoted “SRC”) showed that c-SRC expression was significantly lower in CRC stages II and IV, compared with normal tissues. The area of the combined “pSRC” peaks P1-P5 (Fig. 4e) was also lower in the CRC samples. Moreover, the ratio of pSRC/SRC (Fig. 4f) was lower in CRC than in normal mucosa, indicating that the level of inactivating pY527 phosphorylation was reduced in the cancer compared with normal tissues. There was no apparent correlation between the decreased levels of pSRC/SRC and *KRAS*/*BRAF* mutation status.

### Decreased level of pERK1, but not expression level, in CRC

**Fig. 5:**
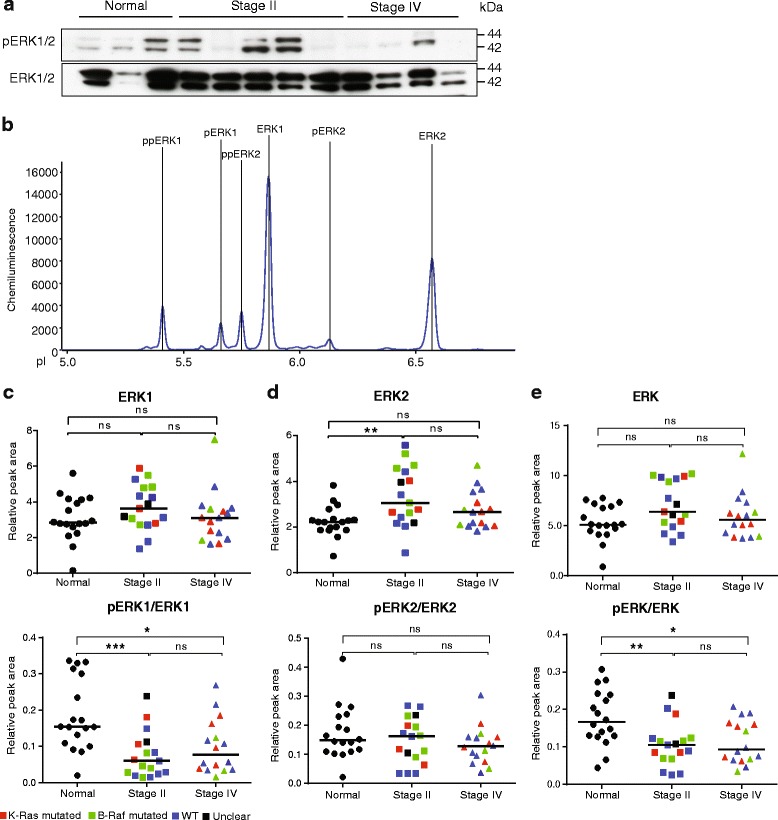
Detection of ERK1/2 total protein and phosphorylated forms by isoelectric focusing. Plots (**c–e**) show values after normalization to HSP70 levels. Symbols in plots: Red; *KRAS* mutated, green; *BRAF* mutated, blue; wild type (WT) with regard to *KRAS* and *BRAF*, black; unclear for *KRAS* and *BRAF*. **a.** Immunoblotting of selected tissue samples with antibodies against pERK1/2 and total ERK1/2 protein. Loading control β2M was same as shown in Fig. 2a. **b.** Representative electropherogram showing ERK1/2 total protein peaks. **c.** Plot of individual peak areas from ERK1 (ppERK1+ pERK1 + ERK1) analyses of normal mucosa and CRC stage II and IV (top) and of pERK1 (ppERK1 + pERK1)/ERK1 peak areas (bottom) after normalization for HSP70 run in parallel. **d.** Plot of normalized ERK2 (ppERK2 + ERK2) protein peaks (top) and pERK2 (ppERK2)/ERK2 (bottom). **e.** Plot of normalized, combined ERK1/2 total protein peaks (top) and combined pERK1/2 over total ERK1/2 peaks (bottom)

Growth factors regulate cell proliferation in the RAS pathway by modifying downstream phosphorylation of the serine/threonine kinases ERK1, on T202/Y204, and ERK2, on T185/Y187. Phosphorylated and nuclearly translocated ERK1/2 catalyze phosphorylation and thereby activation of a range of nuclear transcription factors [37, 38]. Immunoblotting for pERK1/2 showed variable expression in normal mucosa, high expression in stage II and lower expression again in stage IV CRC. The levels of pERK1/2 were variable over the panel of immunoblotted samples (Fig. 5a). Isoelectric focusing on the other hand resolved total ERK1/2 into six major peaks representing both phosphorylated and non-phosphorylated ERK isoforms (Fig. 5b). Using a combination of antibodies reactive with both ERK1 and ERK2, antibodies specifically recognizing only one of the two, and, dephosphorylation by lambda phosphatase, the identity of each peak could be mapped (Fig. 5b). Quantification of the normalized peak areas showed no difference in expression levels of ERK1 between normal mucosa and cancer stage II and IV. However, accumulation of pERK1 decreased in the CRC samples compared to the normal tissue resulting in a significantly decreased pERK1/ERK1 ratio (Fig. 5c). Although ERK2 levels increased in the CRC samples, the pERK2/ERK2 ratios remained unchanged (Fig. 5d). The decrease in pERK1 levels dominated over the increase in pERK2 levels, as a cross-reactive pERK1/2 antibody also showed lower phosphoprotein levels in the cancer samples (Fig. 5e).

### Computational selection of proteins to distinguish CRC from normal tissue

Since individual pathways associated with epithelial cell proliferation showed a very complex pattern in the CRC tissues, we conducted a computational search for combinations of proteins from several pathways that would allow for the discrimination of normal tissue samples from CRC. The overlap between the convex hulls of the data points from normal tissue and CRC stage II or stage IV was examined for every possible combination of up to three features. In addition to the measured 23 different variants (represented by individual peaks in the electropherograms shown in Figs. 1, 2, 3, 4 and 5) for EGFR, AKT, p70S6K, PLCγ1, c-SRC, ERK1, ERK2, and MEK1/2 (see Additional file 1: Figure S2 for MEK1/2 analyses), we also included 15 features constructed as the sum of phosphorylated or non-phosphorylated forms of the seven proteins and their ratios. For detailed description on computational analyses and machine learning see Additional file 1: Figure S3; Characteristics of the data set and errors.

**Fig. 6:**
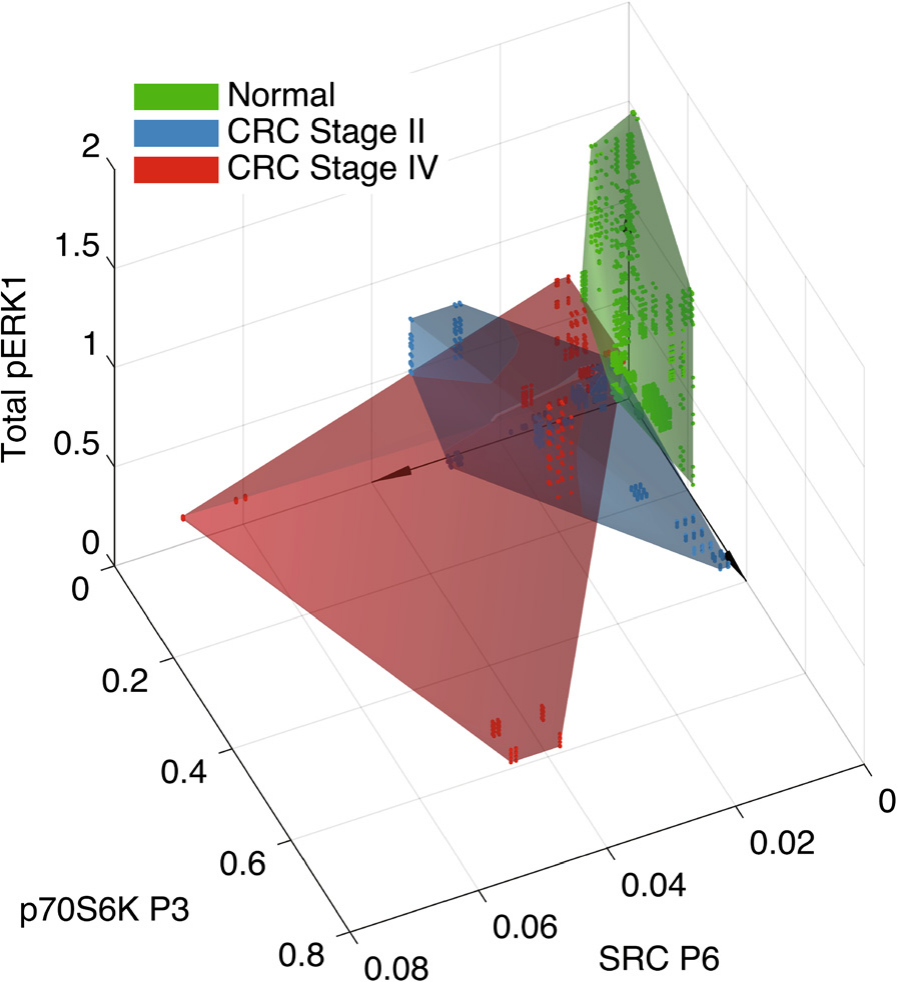
Convex hulls separating normal, CRC stage II and IV tissues. Convex hulls of the sets of all data points of each tissue class representing total pERK1 (ppERK1 + pERK1) peaks, SRC P6 and p70S6K P3 allowed separation of normal tissues (green) from CRC stage II (blue) and stage IV (red). Each dot represents a computationally analyzed data point

In mathematics, the convex hull of a set is the minimal convex set that covers all points in the set. Applied in this context, the convex hull represents the region in protein space that encompasses all observations for either one of the cancer stage or the normal tissue. As shown by the minimal overlap of the convex hulls in Fig. 6, the combination of total pERK1, SRC peak 6 and p70S6K peak 3, separated normal tissue from CRC II and CRC IV. In other words, these three patterns yield a “signature” that was distinct for normal and cancer tissue and measurement of these proteins was sufficient for classification of a tissue sample as normal or CRC. Only one CRC stage IV sample fell within the convex hull of the normal tissues. The convex hulls of the two CRC stages overlapped implying that the combination used (pERK1, SRC peak 6 and p70S6K peak 3) was not appropriate for classification of the disease stage. Monte Carlo simulations revealed that the separation of the non-cancer versus cancer sets was highly unlikely to occur by chance (*p*-value <10^-6^; multiple hypothesis corrected *p*-value <10^-2^). Thus, with this strategy, a unique signature for normal tissue versus cancer tissue was obtained.

## Discussion

Substantial research efforts over the last decades have resulted in increased understanding of CRC mutations and molecular consequences; still, due to the complexity of the tumor biology and the heterogeneity of the cancer, CRC remains a fatal disease. Here, we show that signaling pathways regulating cell survival and proliferation were differently regulated in CRC tissues compared to normal mucosa. Expression of ERK1 and SRC appeared significantly suppressed in CRC tissues compared with normal mucosa while expression of AKT and PLCγ1 were upregulated. See Table 1 for a summary of the pattern of proliferative CRC signaling identified in this study.

**Table 1:** Summary of changes in signaling components between normal and CRC tissues

Signaling component	Normal	CRC II	CRC IV	Comment
EGFR	+	+	+	Similar levels in benign, CRC II and IV.
pAKT/AKT	+	+	+	Total levels of pAKT and AKT upregulated in CRC but pAKT/AKT ratios were similar in the different samples.
p70S6K	+	+	+	Similar levels in benign, CRC II and IV.
PLCγ1	-	+++	+ +	Low or no PLCγ1 expression in benign samples and higher levels in CRC. pPLCγ1 was not detected in any samples.
SRC pY527/SRC	+++	+	+	Low or no SRC pY418 in all samples. Lower SRC pY527/SRC ratios in CRC II and IV compared to benign.
pERK1/ERK1	+++	+	+	Reduced pERK1/ERK1 ratio in CRC II and IV compared to benign.
pERK2/ERK2	++	++	++	Similar ratios benign, CRC II and IV.
MEK1/2	+	+	+	Similar levels in benign, CRC II and IV.

+, ++ and +++ indicate different relative levels or ratios for a particular signaling component when comparing normal, CRCII and CRCIV samples, and should not be applied to compare levels/ratios between the different signaling components

Signaling was analyzed using capillary isoelectric focusing, which we found to be superior to conventional immunoblotting in sensitivity and resolution. After loading of samples and antibodies, the processing was robotized, resulting in highly reproducible and sensitive detection. For example, ERK1/2 protein was detected in 2.5 ng of CRC lysate per capillary (corresponding to 6.25 μg/ml total lysate). Moreover, protein variants, phosphorylated at different residues, could be separated and quantified independently. For ERK1/2 proteins, six of the isoforms (pERK1, ppERK1, ERK1, pERK2, ppERK2, ERK2) could be identified and quantified in relation to the house keeping proteins analyzed in parallel. In comparison, conventional immunoblotting run on the same samples required much more protein for each analysis. It often failed to resolve protein phospho-variants and reproducibility was low, in part due to problems with transfer of proteins to the filter. Ongoing efforts include adapting the isoelectric focusing protocol for the detection of signal transducers in formalin-fixed, paraffin-embedded samples to make the procedure applicable in clinical routines.

Using the isoelectric focusing strategy, several important observations were made that can be related to earlier reports on CRC signaling (see also summary in Table 1):

I) AKT: In agreement with our findings on increased AKT protein expression in the CRC tissues, colorectal adenomas and carcinomas frequently overexpress AKT [39] at an early stage in the disease. Moreover, other components in the PI3K/AKT pathway are affected in CRC. The most common event is a loss of expression of, or mutation in PTEN, which occurs in close to 50 % of the premalignant lesions [40].
II) PLCγ1: Studies on a limited number of CRC samples showed increased PLCγ1 protein levels whereas other PLC family members, PLCβ1 and PLCδ1, remained unaffected [41]. However, whether the increased protein levels are accompanied by increased phospholipase activity in CRC remains unclear. Phosphorylation of PLCγ1 is known to induce its catalytic activity however, we failed to detect phosphorylated PLCγ1 in the CRC samples studied here.
III) c-SRC is a key signal transducer whose activity may initiate most, if not all, other pathways related to cell proliferation [42], and the expression and activity of c-SRC have been associated with CRC progression [15, 16, 42]. However, in several studies, c-SRC activity has been analyzed using an in vitro immune complex kinase assay on cell lines, rather than on clinical samples [43–45]. The lack of pY418 phosphorylated c-SRC and the decrease in expression in disease shown here (Fig. 4), indicate that c-SRC does not drive CRC tumor cell proliferation. Also, pathways potentially induced as a consequence of c-SRC activation in CRC, such as the Scatter factor/c-Met pathway, may not be crucial [46]. c-SRC kinase activity is regulated by tyrosine phosphorylation/dephosphorylation. We detected c-SRC pY527 in all samples, although the amount decreased in disease. As there was no parallel increase in c-SRC pY418, it appears that overall, there is limited c-SRC activity in CRC. The decrease in pY527 levels may depend on phosphatase activity with c-SRC being dephosphorylated *e.g.* by the tyrosine phosphatase PTPRO [47]. Apart from the well characterized positive regulatory pY418 and negative regulatory pY527, there are other phosphorylation sites in c-SRC including pS17 and pY215 whose functions have remained unclear [36]. The many phospho-SRC peaks identified in the isoelectric focusing indicate that, in CRC, c-SRC can become modified at yet additional sites. However, as the critical pY418 is lacking, it is questionable whether c-SRC is a suitable target for CRC therapy. Another complicating aspect of studying c-SRC’s role in cancer biology is the high degree of structural relatedness with other SRC family tyrosine kinases (SFKs), first and foremost the ubiquitously expressed FYN and YES. Thus, we cannot exclude that c-SRC, YES, and FYN phosphoproteins may all have been detected by the c-SRC reagents used here, due to the highly conserved phosphorylation sites in all three members. Overall, insight on the role of the different SFKs in CRC is lacking.
IV) ERK1/2: Aberrant colon crypt foci, which are believed to predict a malignant process, were analyzed using a similar methodology to that applied in this study, revealing elevated levels of both pERK1 and pERK2 irrespective of KRAS and BRAF mutation status [48]. ERK1 and ERK2 are highly related structurally and are largely co-regulated and indeed, in many aspects, redundant. However, ERK2, but not ERK1, has been shown to contribute to RAS-induced oncogenic signaling [49], and yet, ERK1 has been implicated in the negative regulation of ERK2 [50]. Therefore, the reduced pERK1 levels in CRC that we describe here may unleash ERK2 activity, resulting in increased oncogenic signaling in primary tumors. Regulation of ERK1/2 signaling is truly complex, with scaffold proteins, including KSR1/2, IQGAP1, MP1, and β-Arrestin1/2, participating in the regulation of the ERK1/2 MAP kinase cascade [26]. Furthermore, ERK1/2 are dephosphorylated by several different phosphatases [51] that may be differently expressed.

Several decades of ambitious basic and clinical research have demonstrated the challenges in identifying reliable biomarkers in cancer. Challenges include the complexity of the primary tumor tissue consisting of, apart from the tumor cells, a range of host-derived endothelial, fibroblast and inflammatory cells; potential differences between the primary tumor and metastasis; and the possibility that biopsies may not be representative. In this study, the proportion of tumor cells ranged from about 30–60 % in most samples, based on the estimation of mutated DNA/total DNA in the samples (data not shown). An important conclusion from the current study is that the combination of several features from the conducted analyses allows a very high confidence in classifying the tissues as normal or cancerous. The particular combination of pERK1, SRC peak 6, and p70S6K peak 3 selected here to distinguish cancer tissue from normal tissue, may or may not indicate convergence of the included pathways in CRC signaling. The main objective of the selection was to allow unbiased diagnosis. Thus, we propose that reliable prognostic and diagnostic biomarkers should be designed using complex patterns rather than a single molecular or genetic marker. For clinical translation, the isoelectric focusing analyses can easily be made routine and scaled up. For example, 96 unique samples could be run in parallel to yield information on three or more selected pathways (by mixing several appropriate antibodies yielding non-overlapping patterns) in a 10 h run in a robotized set-up. Combined with the powerful computational evaluation to identify sets of signaling components showing significant characteristics, this strategy could prove to be clinically feasible for diagnostic purposes beyond the treatment of CRC. Based on the results obtained this far, we predict that the measurement of seven protein forms (i.e. selected peaks from the electropherograms) would be sufficient for the correct classification of both non-cancerous versus cancerous tissue as well as for the CRC grade. Moreover, analysis of a larger cohort of samples, combined with information on chosen therapy and disease outcomes, would allow the use of supervised learning for identification of clinically relevant subtypes.

## Conclusions

- Highly sensitive robotized isoelectric focusing was established and a wide range of signal transduction pathway antibodies were validated. The set-up was shown to allow detection of signaling status also in extremely scarce samples, not amenable to conventional analysis performed in parallel.
- The study revealed dysregulated signal transduction in several proliferative pathways in human colorectal cancer tissue, which did not correlate with the mutation status.
- Computational analysis was used to identify signal activities consisting of three components that, when combined, could accurately identify normal mucosa from cancer.

The study suggests that such combinations of different signalling activities could serve as predictive or prognostic complex biomarkers.

## Additional file

Additional file 1:
**Figure S1.** Validation of antibodies used in the study by conventional immunoblotting. All antibodies showed immunoreactivity with the expected molecular species, in conventional immunoblotting on endothelial lysates. **Figure S2.** Detection of MEK1/2 protein by isoelectric focusing. There was no significant difference in MEK protein expression between normal, CRCII and CRCIV tissues. Detailed description of computational analyses; “Characterization of the data set and errors”. **Figure S3.** Distribution function for data subsets by Monte Carlo simulation. (DOCX 1215 kb)

## Abbreviations

CCD: Charge-coupled device
CRC: Colorectal cancer
EGFR: Epidermal growth factor receptor
ECL: Enhanced chemiluminescence
ERK: Extracellular regulated kinase
HSP70: Heat shock protein 70
IP3: Inositol 1,4,5-trisphosphate
MEK: Mitogen-activated protein kinase kinase
mTOR: Mammalian target of rapamycin complex
PDK1: Phosphatidylinositol-dependent kinase 1
pI: Isoelectric point
PIP2: Phosphatidylinositol 4,5 bisphosphate
PLCγ1: Phospholipase Cγ1
PTEN: Phosphatase and tensin homolog
WT: Wild type
