# Supplementary material for: High sensitivity isoelectric focusing to establish a signaling biomarker for the diagnosis of human colorectal cancer
Source: BMC Cancer. 2016 Aug 25;16(1):683. doi: 10.1186/s12885-016-2725-z (PMC5000422; doi:10.1186/s12885-016-2725-z)
Supplement: Additional file 1: — Figure S1. Validation of antibodies used in the study by conventional immunoblotting. All antibodies showed immunoreactivity with the expected molecular species, in conventional immunoblotting on endothelial lysates. Figure S2. Detection of MEK1/2 protein by isoelectric focusing. There was no significant difference in MEK protein expression between normal, CRCII and CRCIV tissues. Detailed description of computational analyses; “Characterization of the data set and errors”. Figure S3. Distribution function for data subsets by Monte Carlo simulation. (DOCX 1215 kb) [file 12885_2016_2725_MOESM1_ESM.docx]

**Supplemental data set**

High sensitivity isoelectric focusing to establish a signaling biomarker for the diagnosis of human colorectal cancer

Narendra Padhan^1)^, Torbjörn E. M. Nordling^1,2,§)^, Magnus Sundström^1)^, Peter Åkerud^3)^, Helgi Birgisson^3)^, Peter Nygren^1)^, Sven Nelander^1)^ and Lena Claesson-Welsh^1)*^

1. Uppsala University, Dept. Immunology, Genetics and Pathology, Rudbeck Laboratory, Uppsala University, 751 85 Uppsala, Sweden
2. Stockholm Bioinformatics Centre, Science for Life Laboratory, Box 1031, 171 21 Solna, Sweden
3. Uppsala University, Dept. Surgical Sciences, Uppsala University, 751 85 Uppsala, Sweden

§ Current address: Dept. of Mechanical Engineering, National Cheng Kung University, No. 1 University Road, Tainan 70101, Taiwan

**Fig. S1, Padhan et al.**

**
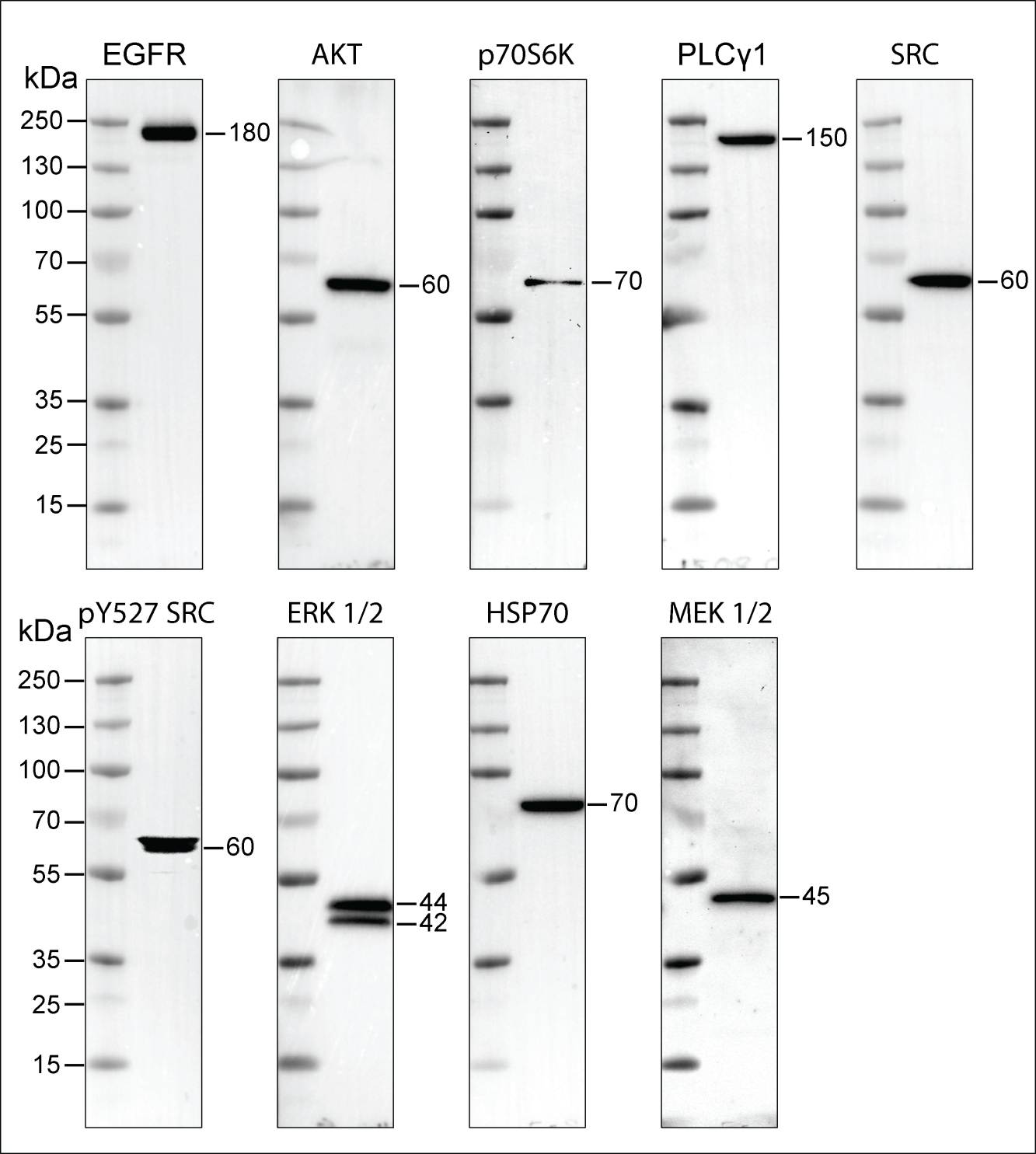
**

**Antibody verification**

All antibodies used for isoelectric focusing were verified by immunoblotting. For each blot, two images, colorimetric and chemiluminescent, were acquired by a ChemiDoc™ MP Imaging System (Bio-Rad). The two images were merged and shown here to assess the protein size and specificity of each antibody. HUVEC cell lysate was used in all blots except for EGFR where A431 cell lysate was used.

**
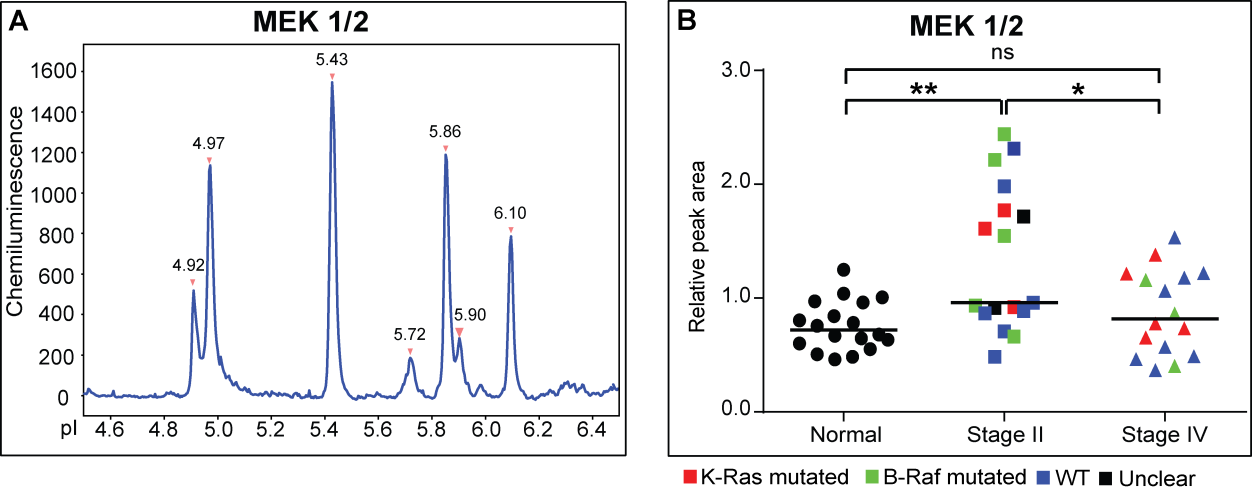
Fig. S2, Padhan et al.**

**Detection of MEK 1/2 protein by isoelectric focusing**

1. Representative electropherogram showing MEK 1/2 total protein peaks.
2. Plot of MEK 1/2 peak areas in samples from normal tissue, CRC stage II and IV biopsies. Values were normalized to HSP70 levels. Symbols in plots: Red; *KRAS* mutated, green; *BRAF* mutated, blue; wild type (WT) with regard to *KRAS* and *BRAF*, black; unclear for *KRAS* and *BRAF*.

**Characteristics of the data set and errors**

The data set contained 30 variables; 23 different activity levels of 7 signal transducers (EGFR, PLCγ1, AKT, 70SK, SRC, ERK, MEK), 3 binary variables indicating mutation in BRAF, KRAS, or wildtype, and 3 binary variables indicating the classification of each sample as normal mucosa, colorectal cancer (CRC) stage II, or CRC stage IV. Measurements for a total of 66 patient samples existed. The total number of data points was 3921 including technical replicates, and 1317 excluding technical replicates.

The null hypothesis of the residuals from both the mean and median of the measurements being normally distributed was rejected by a Lilliefors’ goodness-of-fit test of composite normality (1), with p-value below 10*^−^*^4^. A considerable number of technical replicates were further away from the mean or median of the measurements than expected for normally distributed data. Moreover, a considerable number of technical replicates was closer to the mean or median of the measurements than expected for normally distributed data, in part due to measurements with only one technical replicate. We thus included residuals of the 1084 measurements with more than one technical replicate that had a range smaller than 0.15 Relative Peak Area (RPA). However, the null hypothesis of the residuals being normally distributed was also in this case rejected by a Lilliefors’ goodness-of-fit test of composite normality, with p-value below 10*^−^*^4^. The measurement errors were also not Cauchy distributed (data not shown). All calculations were done in Matlab R2014a using the Statistics Toolbox.

Certain transformations of the observed features, such as the ratio of phosphorylated and non-phosphorylated forms of a protein or the sum of all forms of the same protein, are common in the biological literature and we therefore constructed 15 additional features of this type (see the results), so that the total number of features investigated became 41. When constructing these we made every possible combination of technical replicates of original measurement with less than 5 replicates, while we only made every possible combination of the minimum and maximum replicate of original measurements with more than 4 replicates.

Since the number of replicates for a majority of the measurements were three or less and the distribution of the measurement errors remained unknown, we based our analysis of differences among the classes on the overlap of sets, formed by extreme values among all replicates for each sample and variable. Considering the relatively small number of samples, 18 normal, 17 CRC grade II, and 16 CRC grade IV, and the unknown distribution of the measurement errors, we decided to represent each class by the convex hull of all technical replicates of measurements of samples belonging to the class in question. A convex set is characterized by containing all intermediary points on the line between any two points in the set, moreover, the convex hull is the smallest convex set that contains a specified set of points (2).

**Feature selection for separation of the classes**

Typically when feature selection is employed, the objective is to find the optimal model for classification or prediction of samples of unknown class. The data set is divided into a training set used for feature selection and estimation of model parameters, and a validation set for evaluation of how well the model performs in classification or prediction. Our objective was to find the subset(s) of proteins that could be used to distinguish between normal and cancer tissue, in order to study how it relates to existing knowledge on the biology of colorectal cancer. We therefore used all samples for feature selection instead of dividing our data into two sets.

To asses the significance of the separation of the classes and measure their overlap, we chose to count the number of samples belonging to another class within the convex hull, in favor of more complicated measures based on the distance of data points to the hull. We focused on differences between normal and cancer samples and chose the test statistic (*T)*: Number of normal samples within the convex hull of the CRC grade II or IV samples plus the number of CRC grade II or IV samples within the convex hull of the normal samples. The strength of this method lies in the ability to inspect it visually and interpret it using two simple concepts–separation between sets and p-values.

We decided to construct convex hulls for the three classes and evaluated our test statistic for every possible combination of up to three features. The number of possible combinations was 10660 for three included features, 820 for two, and 41 for one. We performed all calculations in Matlab R2014a using the built-in functions convhulln and inpolygon, as well as inhull by John D’Errico, see http:// [www.mathworks.com/matlabcentral/fileexchange/10226-i](http://www.mathworks.com/matlabcentral/fileexchange/10226-i)[nhull](http://www.mathworks.com/matlabcentral/fileexchange/10226-inhull). All possible combinations together with their test statistic and number of included samples are deposited at [http://datadryad.org](http://datadryad.org/).

**Significance of separation of the classes**

To assess the significance of separation of the classes, we needed to test the hypothesis *H*0: the abundance of the proteins included in the selected subset has no relation to the class of the included samples. For this we needed the cumulative distribution function (CDF), *F* (*t*), of our test statistic *T* for random data with the same properties as the recorded data but no relation between the abundance levels and the classes. The CDF tells us the probability of observing a value smaller or equal to *t*, *P* (*T ≤ t*), i.e. the p-value of *t*. If the probability is smaller than the required significance level, then we can reject the null hypothesis *H*0. The Monte Carlo simulations used to generate the CDF are presented elsewhere (3).

The calculated CDF for subsets of one to six features, i.e. p-values for rejecting *H*0, together with corresponding p-values corrected for multiple testing, are shown in Fig. S3. We also included the p-values corrected for multiple testing, since we, for each number of included features, searched for the best combination, minimizing the test statistic. The two p-values have different connotations. The first gives the probability of the observed separation of the normal and CRC sets, i.e. test statistic, when the protein levels have no relation to the three classes (when the null hypothesis is true), while the second one gives the probability of the observed separation being within the number of tested cases when the null hypothesis is true.

The second one controls the so-called, family-wise error rate of rejecting the null hypothesis when it is true. In both cases the probability to observe separated sets increases with the number of features included in the subset due to the number of samples becoming more scarce, relative to the number of dimensions of the space. If the number of samples would be increased, the curves would shift to the right (Fig. S3).

For subsets containing three features the p-value was below 10*^−^*^4^ as long as the test statistic was below 5. 13 different subsets had *T <* 5. The top one–SRC P6, p70S6K P3, and Total pERK1– with *T* = 1 had a p-value below 10*^−^*^6^ and corrected p-value below 7X10*^−^*^3^.

**References**

1. Lilliefors HW: On the Kolmogorov-Smirnov test for normality with mean and variance unknown. J. Am. Stat. Assoc. 1967, **62**, 399-402.
2. Boyd SP, and Vandenberghe L: Convex optimization (Cambridge University Press, 2004) xiii, 716 p.
3. Nordling, TEM, Padhan N, Nelander S, and Claesson-Welsh L: Identification of biomarkers and signatures in protein data. *e-Science (e-Science), 2015 IEEE 11th International Conference on*, Munich, 2015, pp. 411-419. doi: 10.1109/eScience.2015.46

**Fig. S3, Padhan et al.**


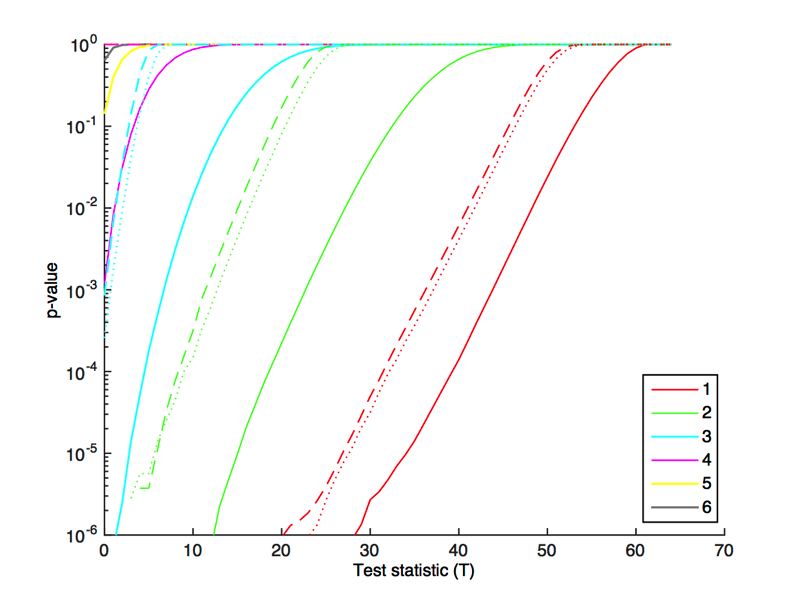


**Distribution function for subsets**

To estimate what constitutes an extreme value of our classification statistic T (defined in the supplementary text), we applied a Monte Carlo simulation. In this simulation, the null distribution of T was simulated by permutation of sample class labels, obtaining a cumulative distribution function (cdf). The cdf for T is dependent on the number of features chosen (1-6) and the feature selection strategy (solid lines: random selection; dashed lines: optimal subset; dotted lines: combinations with more than 45 samples).
